# Mitochondrial dysfunction contributes to the senescent phenotype of IPF lung fibroblasts

**DOI:** 10.1111/jcmm.13855

**Published:** 2018-09-26

**Authors:** Michael Schuliga, Dmitri V Pechkovsky, Jane Read, David W Waters, Kaj E C Blokland, Andrew T Reid, Cory M Hogaboam, Nasreen Khalil, Janette K Burgess, Cecilia M Prêle, Steven E Mutsaers, Jade Jaffar, Glen Westall, Christopher Grainge, Darryl A Knight

**Affiliations:** ^1^ School of Biomedical Sciences and Pharmacy University of Newcastle Callaghan New South Wales Australia; ^2^ Hunter Medical Research Institute New Lambton Heights New South Wales Australia; ^3^ Department of Anesthesiology Pharmacology and Therapeutics University of British Columbia (UBC) Vancouver British Columbia Canada; ^4^ Department of Pathology and Medical Biology University of Groningen University Medical Center Groningen Groningen Research Institute of Asthma and COPD and KOLFF Institute Groningen Netherlands; ^5^ Department of Medicine Cedars‐Sinai Los Angeles California; ^6^ Department of Respiratory Medicine UBC Vancouver British Columbia Canada; ^7^ Institute for Respiratory Health University of Western Australia Nedlands Western Australia Australia; ^8^ Centre for Cell Therapy and Regenerative Medicine School of Biomedical Sciences University of Western Australia Crawley Western Australia Australia; ^9^ Allergy, Immunology and Respiratory Medicine Alfred Hospital Prahran Victoria Australia; ^10^ School of Medicine and Public Health University of Newcastle Callaghan New South Wales Australia

**Keywords:** cyclin‐dependent kinase inhibitors, fibroblasts, idiopathic pulmonary fibrosis, mechanistic target of rapamycin complex 1, mitochondria, peroxisome proliferator‐activated receptor gamma coactivator 1‐alpha, rapamycin, reactive oxygen species and mitoTEMPO

## Abstract

Increasing evidence highlights that senescence plays an important role in idiopathic pulmonary fibrosis (IPF). This study delineates the specific contribution of mitochondria and the superoxide they form to the senescent phenotype of lung fibroblasts from IPF patients (IPF‐LFs). Primary cultures of IPF‐LFs exhibited an intensified DNA damage response (DDR) and were more senescent than age‐matched fibroblasts from control donors (Ctrl‐LFs). Furthermore, IPF‐LFs exhibited mitochondrial dysfunction, exemplified by increases in mitochondrial superoxide, DNA, stress and activation of mTORC1. The DNA damaging agent etoposide elicited a DDR and augmented senescence in Ctrl‐LFs, which were accompanied by disturbances in mitochondrial homoeostasis including heightened superoxide production. However, etoposide had no effect on IPF‐LFs. Mitochondrial perturbation by rotenone involving sharp increases in superoxide production also evoked a DDR and senescence in Ctrl‐LFs, but not IPF‐LFs. Inhibition of mTORC1, antioxidant treatment and a mitochondrial targeting antioxidant decelerated IPF‐LF senescence and/or attenuated pharmacologically induced Ctrl‐LF senescence. In conclusion, increased superoxide production by dysfunctional mitochondria reinforces lung fibroblast senescence via prolongation of the DDR. As part of an auto‐amplifying loop, mTORC1 is activated, altering mitochondrial homoeostasis and increasing superoxide production. Deeper understanding the mechanisms by which mitochondria contribute to fibroblast senescence in IPF has potentially important therapeutic implications.

## 1 INTRODUCTION

Idiopathic pulmonary fibrosis (IPF) is a lethal disease of unknown aetiology that largely presents in the elderly. Although the pathogenesis of IPF is unclear, it is considered to be a consequence of dysregulated repair responses emanating from an injured epithelium.^1^ Epithelial cell hyperplasia and myofibroblast/collagen accumulation in lung parenchyma lead to tissue structural defects that impair gas exchange.^2^ Until recently, effective treatment options for IPF were limited to lung transplantation. However, both pirfenidone and nintedanib have been shown to reduce the rate of decline in lung function and increase progression free survival in IPF, albeit with significant side effects.^3^, ^4^ These positive developments have encouraged renewed interest in identification of alternative and complementary drug targets.

Cellular senescence follows a DNA damage response (DDR) and subsequent induction of p53‐p21^CIPI^ and/or p16 ^INK4a^‐pRB signalling,^5^ both of which lead to cell‐cycle arrest. Senescent cells are also resistant to apoptosis, evade immune surveillance and acquire a senescence‐associated secretory phenotype (SASP).^6^, ^7^, ^8^ There is mounting evidence to support an important contribution of senescence in IPF. For example, mutations in genes encoding components of the telomerase complex that accelerate replicative senescence are linked to IPF and senescent markers are detected in the lung of IPF patients.^9^, ^10^, ^11^, ^12^ Furthermore, senescent prone mice are more susceptible to experimental pulmonary fibrosis, whereas the targeting of senescent cells is protective.^6^, ^13^ Recent evidence suggests that the senescence of type II alveolar epithelial cells (AECIIs) in IPF impairs re‐epithelialization following injury, triggering a cascade of events that lead to fibrosis.^14^, ^15^ Senescent lung fibroblasts (LFs) are also highly likely have a role in IPF, exhibiting myofibroblast‐like characteristics (ie*,* increased α‐smooth muscle actin expression) and a highly activated secretome.^6^, ^11^, ^12^, ^16^ In IPF, a failure to eliminate senescent fibroblasts by apoptosis or immune cell clearance is postulated to impede wound resolution and contribute to disease progression.^11^, ^12^

Mitochondria are double membrane‐bound organelles responsible for energy production. Mitochondrial integrity is maintained through the coordination of several processes such as biogenesis and mitophagy, which have been collectively referred to as mitochondrial quality control (MQC).^17^ Dysfunctional MQC and inflammation are key signatures of ageing and several ageing‐related diseases. As part of the mitochondrial energy generating process involving the electron transport chain (ETC), high‐energy electrons leak and generate reactive oxygen species (ROS). Mitochondrial DNA (mtDNA) is highly sensitive to oxidative damage because of its proximity to the ETC and ROS production, lack of chromatin structure and a reduced capacity for repair.^18^ As such, mtDNA damage and subsequent mutations can result in mitochondrial dysfunction, including a collapse in the mitochondrial membrane potential,^19^ which in turn contributes to further ROS production, that ultimately leads to dysmorphic and impaired mitochondria. Increased mtDNA levels and mass occur in a number of ageing organs and tissues, including the lung, possibly as an adaptive mechanism to compensate for diminished mitochondrial output.^20^

Sustained increases in ROS as a result of mitochondrial dysfunction are thought to reinforce the DDR, and senescence as a consequence.^21^, ^22^, ^23^ Senescent LFs from IPF patients (IPF‐LFs) have recently been reported to exhibit features of mitochondrial dysfunction, including disrupted cristae and a diminished capacity for oxidative phosphorylation.^24^ However, the consequences of these mitochondrial perturbations on superoxide production have not been evaluated, nor any association between senescence and mitochondrial dysfunction. In this study, we characterize for the first time the relationship between the DDR, senescence and mitochondrial dysfunction in IPF‐ and Ctrl‐LFs involving mitochondrial superoxide. We provide evidence that in IPF‐LFs, an auto‐amplifying loop exists involving mitochondrial‐derived ROS which perpetuates senescence, and that the disruption of this cycle has potential important therapeutic implications. Increased activation of the mechanistic target of rapamycin complex 1 (mTORC1) and subsequent alterations in mitochondrial homoeostasis mediate the increased ROS production that follows a DDR and contributes to senescence persistence in LFs.

## 2 METHODS

### 2.1 Cell culture

LF cell cultures were established as described previously^25^ using lung tissue resections from patients at the John Hunter Hospital (New Lambton Heights, Australia) under ethical approval from the Hunter New England Human Research Ethics Committee (16/07/20/5.03) following guidelines from the National Health and Medical Research Council (NHMRC, Australia). All patients had provided written, informed consent. Cell cultures were also obtained from the Alfred Lung Fibrosis BioBank (Alfred Hospital, Melbourne, Australia) under ethical approval from the Alfred Health Ethics Committee (#336/13) following NHMRC guidelines. Donors were classified as either IPF or Control (Ctrl). IPF patients were accurately phenotyped by respiratory clinicians in regard to underlying diagnosis and disease severity, with patient characteristics listed in Table S1. Age‐matched controls were either patients with no evidence of interstitial lung disease undergoing routine thoracic surgical procedures or lung transplant donors. LFs in culture, established from separate patients/donors were used at an early passage (1‐6) to minimize complications with replicative senescence. For experiments directly comparing Ctrl and IPF‐LFs, the average age of the donors or patients is shown in Table S2. Cells were grown in Dulbecco's modified Eagle's medium (DMEM) containing high glucose (4.5 g/L), L‐glutamine (2 mmol L^−1^), sodium pyruvate (1 mmol L^−1^), non‐essential amino acids (1% v/v, Sigma) and heat‐inactivated foetal calf serum (FCS) (10% v/v) at 37°C in air containing 5% CO_2_ for senescence characterization at baseline. For chemical‐induced senescence experiments, cells were replenished in serum‐reduced DMEM containing 0.4% v/v FCS for 24 hour before addition of etoposide (10 μmol L^−1^), rotenone (100 nmol L^−1^) or the appropriate volume of DMSO as vehicle control. Therapeutics, including rapamycin (100 nmol L^−1^), mitoTEMPO (1 μmol L^−1^) or N‐acetyl cysteine (NAC, 2 mmol L^−1^), were added 30 minute before etoposide.

### 2.2 Immunofluorescence

Phosphorylated p53 and histone H2A.X (γH2A.X) in nuclei and CoxIV in mitochondria were detected by immunofluorescence. Cells grown in 48‐well plates were fixed with 4% w/v formaldehyde in PBS for 10 minute before blocking and permeabilization with 0.15% v/v Triton X‐100, 10% v/v goat serum and 1% w/v BSA in PBS for 10 minute. Cells were then incubated with anti‐phospho‐p53 (Ser15) (#9284, Cell Signaling Technology) or ‐phospho‐γH2A.X (Ser139) (#9718, Cell Signaling Technology) rabbit polyclonal IgG and anti‐CoxIV (#11967 Cell Signaling Technology) mouse monoclonal IgG, overnight at 4°C. After washing, cells were incubated with Alexa Fluor 555 anti‐rabbit‐ and 488 anti‐mouse conjugates (#4413 and #4408, respectively, Cell Signaling Technology) for 1 hour at room temperature. All antibodies were used at a 1 in 200 dilution. Cells were counterstained with DAPI (1 μg/mL, Sigma) and mounted in 70% v/v glycerol. Fluorescent images of cells were taken at 100× magnification using a Nikon Eclipse Ti‐U fluorescence microscope.

### 2.3 Senescence‐associated β‐galactosidase detection

For senescence‐associated β‐galactosidase (SA‐β‐Gal) staining, sub‐confluent cell cultures in 12‐well plates were fixed and stained using a commercial kit (Cell Signaling Technology) according to the manufacturer's instructions. Cells were imaged using an Olympus IX51 inverted microscope. For quantitation, blue stained cells were visualised by light microscopy, and counted in random fields at 100× magnification. Positive cells were expressed as a percentage of total cells, counted in the same fields by phase‐contrast microscopy.

### 2.4 ROS and mitochondrial stress measurements

To detect intracellular ROS or mitochondrial superoxide levels, cells were replenished in phenol‐red free DMEM before staining with 2’,7’‐dichlorodihydrofluorescein diacetate (DCFDA, 5 μmol L^−1^, Sigma) or MitoSOX (1 μmol L^−1^, Molecular Probes), respectively, for 20 minute at 37°C. Mitochondrial stress and mass were evaluated by staining cells with 10‐n‐Nonyl‐Acridine Orange (NAO, 5 μmol L^−1^, Sigma) and MitoTracker Green (0.5 μmol L^−1^, Molecular Probes), respectively. Stained cells grown in 96‐well plates were analysed using a FLUOstar OPTIMA microplate reader (BMG Labtech). The excitation and emission wavelengths for DCFDA, MitoTracker Green and NAO were 485 and 520 nm, respectively, whereas for MitoSOX, they were 485 and 590 nm, respectively. For fluorescence microscopy, stained cells grown in 48‐well plates were counterstained with DAPI (1 μg/mL) before imaging using a Nikon Eclipse Ti‐U inverted microscope at 100× magnification.

### 2.5 siRNA transfection

Cells grown in 12‐ or 24‐well plates were transfected with 20 nM RNA short interference (siRNA) duplex oligonucleotides using RNA*i*Max Lipofectamine (Invitrogen, CA, USA). Prior to transfection, cells were replenished in antibiotic‐free serum containing DMEM, before incubation with Lipofectamine‐siRNA complex for 6 hour. The media was then replaced with DMEM containing 10% FCS. PGC‐1α and control siRNA (Sigma‐Aldrich, MO, USA) were used in the study. Two sequences of PGC‐1α siRNA were used at a 1:1 ratio: 5’‐GCUACUAUGAGCACGUGAA [dT] [dT]‐3’; and 5’‐GCUGUAACACUUCUUAUUA [dT] [dT]‐3’.

### 2.6 ELISA and protein assays

Levels of interleukin‐6 (IL‐6), IL‐8, monocyte chemoattractant protein‐1 (MCP‐1 or CCL2), regulated on activation, normal T cell expressed and secreted protein (RANTES or CCL5) and insulin‐like growth factor binding protein‐5 (IGFBP5) in LF supernatants were measured by specific sandwich enzyme‐linked immunosorbent assays (ELISA) using commercial kits (RnDSystems, MN, USA) as according to the manufacturer's instructions. Protein concentration in cell lysates was measured using the BCA assay kit (Thermo Scientific).

### 2.7 nCounter multiplex digital mRNA profiling

Fibroblast inflammatory gene expression was profiled using the nCounter Human Inflammation v2 Panel (NanoString Technologies, Seattle, WA), comprising 249 human inflammatory genes and additional housekeeping genes (http://www.nanostring.com/products/gene-expression-panels/ncounter-inflammation-panels). For each sample, RNA (100 ng) was hybridised to the inflammatory gene expression code set before purification and immobilization to nCounter cartridges using the nCounter Prep Station (NanoString Technologies). Cartridges were scanned using the nCounter Digital Analyzer and subsequent data analysed with nSolver software (NanoString Technologies). Raw data of each gene (digital counts) were normalized to housekeeping genes (CLCT1, GUSB, HPRT1 and TUBB) before the ratio of the IPF versus Ctrl samples was computed. Heat maps of differentially regulated genes were generated online using Morpheus software (https://software.broadinstitute.org/morpheus/).

### 2.8 PCR analysis

Real time polymerase chain reaction (PCR) was conducted to quantify mRNA and mitochondrial DNA. RNA and DNA were purified from cells using RNeasy and QIAamp DNA mini spin columns (Qiagen), respectively. RNA was reverse transcribed into cDNA using the iScript Advanced cDNA kit (Bio‐Rad). DNA was amplified by PCR using the iTaq Universal SYBR Green Supermix (Bio‐Rad) in an ABI Prism 7500HT sequence detection system (Applied Biosystems) with the relevant PCR primers (Table S3). For RNA quantitation, the threshold cycle (CT) value determined for each gene of each sample was normalized against that obtained for the internal controls, GAPDH or 18S rRNA. The level of mRNA for a particular gene is proportional to 2^−(ΔCT)^, where ΔCT is the difference between the CT values of the target and control. Similarly, mitochondrial DNA content relative to nuclear DNA was calculated by subtracting the CT value of the nuclear gene, B2‐microglobulin from the CT value of the mitochondrial gene, tRNA‐Leu (UUR).^26^

### 2.9 Immunoblotting

Cell lysates (4 μg protein) were subjected to SDS polyacrylamide gel electrophoresis (SDS‐PAGE) using 4%‐15% Mini‐Protean TGX 15 well stain free gels (Bio‐Rad). Fluorescent detection of protein after electrophoresis was imaged using a Bio‐Rad Gel Doc imaging system. Gels were then electroblotted as described previously^27^ before membranes were blocked with 1.5% v/v BSA and 2.5% skim milk in TBS‐T (10 mmol L^−1^ Tris; 75 mmol L^−1^ NaCl; 0.1% Tween‐20; pH 7.4) for 1 hour. Membranes were incubated overnight at 4°C with rabbit polyclonal IgGs raised against NF‐κB p65 (1:4000, Abcam) or phosphorylated‐p70SK (1:1000, Cell Signaling Technology). Blots were washed with TBS‐T prior to incubation with goat anti‐rabbit (Chemicon) IgG‐horse raddish peroxidase conjugate (diluted 1:4000) for 1 hour at room temperature. Antigen was detected by enhanced chemiluminescence (Amersham Biosciences, UK) using a Bio‐Rad Gel Doc imaging system.

### 2.10 Statistical analysis

Data are presented as box and whiskers plots where *n* represents individual experiments conducted using cells from separate donors. Data were analysed by *t*‐test, Mann‐Whitney U test or analysis of variance (ANOVA) (Graphpad Prism 5.0, Graphpad, San Diego, CA), where appropriate. For nCounter gene analysis, the *P* values of the ratio of IPF vs Ctrl samples for each gene were calculated using nSolver software (NanoString Technologies). A value of *P* < 0.05 was considered to be statistically significant.

## 3 RESULTS

### 3.1 IPF‐LFs exhibit senescence‐like characteristics

**Figure 1:**
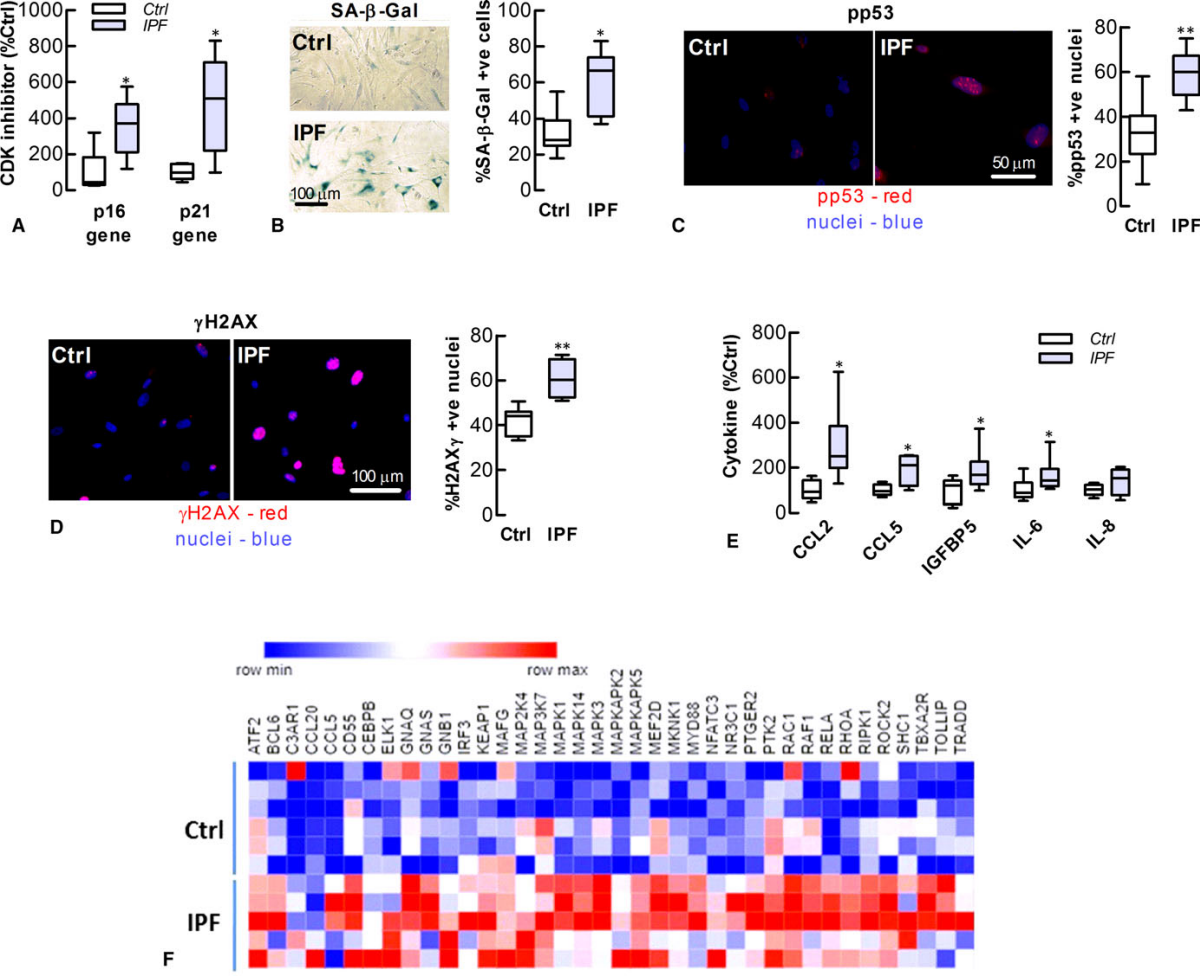
Lung fibroblasts of IPF patients exhibit senescence‐like characteristics. IPF‐ and Ctrl‐LFs were examined for senescence phenotype markers. (A) Baseline levels of CDK inhibitor (p16 and p21) mRNA (n = 5‐6). Gene (mRNA) data were normalized to GAPDH with levels of Ctrl‐LFs (2^−Δ^ ^CT^ x 10^3^) for p21 and p16 being 167 ± 30 and 0.031 ± 0.015, respectively. (B) *Left* SA‐β‐Gal activity in LFs as detected by cytochemical staining (blue). *Right* Quantitative data (n = 7) (C) Immunofluorescence detection of activated p53 (pp53) *Left* Images showing accumulation of pp53 (red) in the nuclei (blue) of IPF‐LFs. *Right* Quantitative data (n = 8‐10). *P < 0.05, compared to Ctrl‐LFs. (D) Detection of phosphorylated γH2A.X. *Left* Immunofluorescence images. *Right* Quantitative data (n = 5‐6). *P < 0.05, compared to Ctrl‐LFs. (E) Levels of SASP cytokine protein in the condition medium of LF cultures. Cytokine data were normalized to total protein with Ctrl‐LF levels for CCL2, CCL5, IGFBP5, IL‐6 and IL‐8 being 87 ± 16, 0.23 ± 0.03, 248 ± 56, 175 ± 36 and 327 ± 35 ng/mg protein, respectively. *P < 0.05, compared to Ctrl‐LFs (n = 5‐9). (F) Heat map showing the up‐regulated genes of the nCounter human inflammatory gene panel; IPF‐ vs Ctrl‐LFs (*P* < 0.05, n = 5‐6). The rows and columns represent samples and inflammatory genes, respectively, whereas colours depict relative gene expression, with blue and red being lower and higher expression, respectively

A composite set of parameters were measured to ascertain the extent of IPF‐LF senescence. The expression of the cell‐cycle arrest proteins p16 and p21 and the percentage of cells positive for SA‐β‐Gal were higher in cultures of IPF‐LFs, compared to the Ctrl‐LFs (Figure 1A and B). Furthermore, a higher percentage of nuclei in the IPF‐LFs exhibited foci of phospho‐p53 (pp53), a DDR marker (Figure 1C). Like p53, phosphorylated histone H2A.X (γH2A.X) rapidly forms complexes at DNA double‐stranded breaks and is a sensitive marker of the DDR. The formation of γH2A.X nuclear foci was also increased in IPF‐LFs, when compared to Ctrl‐LFs (Figure 1D). An important pathological feature of senescent fibroblasts is the SASP and while the SASP of IPF‐LFs has previously been described, its characterization was limited to the gene expression of a few inflammatory genes.^24^ In this study, the levels of IL‐6, CCL2, CCL5 and IGFBP5 in IPF‐LF conditioned medium were shown to be higher than Ctrl‐LF conditioned medium (Figure 1E). Using nanostring technology, 43 inflammatory genes were differentially regulated between IPF‐ and Ctrl‐LFs (*P* < 0.05), with 39 increased in IPF (Figure 1F). This included the expression of CCL5 and CCL20, CCAAT/enhancer‐binding protein‐β (C/EBPβ), nuclear factor‐kappa‐B p65 (NF‐κB or RELA), MyD88 and numerous mitogen‐activated protein kinases (MAPKs) (graphical data of selected genes are presented in Figure S1A). In addition, the levels of NF‐κB, a pivotal regulator of SASP cytokine expression, were also higher in IPF‐LFs (Figure S1B). Collectively, these data confirm that LFs of IPF patients are more senescent‐like than Ctrl‐LFs, corresponding with an intensified DDR and SASP.

### 3.2 IPF‐LFs display mitochondrial dysfunction

**Figure 2:**
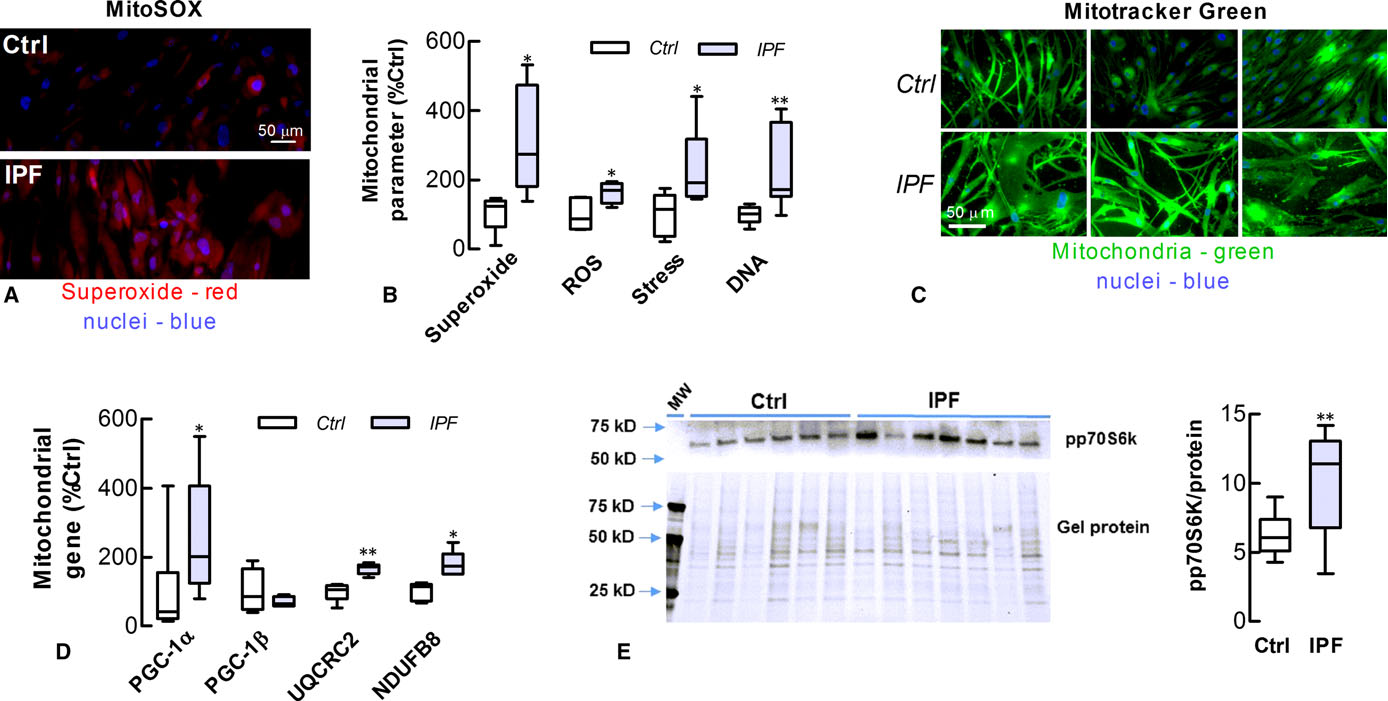
Lung fibroblasts of IPF patients exhibit mitochondrial dysfunction. (A) Superoxide production as detected using the MitoSOX fluorophore (Red) in IPF and Ctrl‐LFs. Nuclei are counter‐stained using DAPI (blue). (B) Levels of mitochondrial superoxide, cellular ROS, mitochondrial DNA and stress (n = 4‐7). Mitochondrial stress was evaluated using the NAO fluorophore and microplate fluorometry. (C) Mitochondrial staining using MitoTracker Green in three separate cultures of IPF‐ and Ctrl‐LFs. (D) Expression of mitochondrial‐associated genes (n = 4‐7). mRNA data was normalized to GAPDH mRNA with Ctrl‐LF levels (2^−Δ^ ^CT^ x 10^3^) for PGC‐1α, PGC‐1β, UQCRC2 and NDUFB8 being 12 ± 7, 368 ± 40, 24 ± 2 and 40 ± 4, respectively. (E) mTORC1 activity was measured by increases in the phosphorylation of the mTORC1 substrate, p70S6k. *Left top* Immunoblot detection of pp70S6K in cell lysates obtained from LFs of separate IPF and Ctrl donors. *Left bottom* Fluorescent detection of total protein in gels before immunoblotting to verify protein loading. *Right* Quantitative data. *P < 0.05, **P < 0.01 compared to Ctrl‐LFs

Evidence is accumulating that mitochondrial‐derived superoxide and its ROS by‐products elicit a DDR to reinforce senescence.^22^, ^23^ Adding further evidence, the levels of mitochondrial superoxide and intracellular ROS in this study were significantly higher in IPF‐LFs than Ctrl‐LFs (Figure 2A and B). IPF‐LFs exhibited other features of mitochondrial dysfunction including higher levels of mitochondrial DNA (mtDNA) and stress (Figure 2B). Fluorescence images of three separate cultures of IPF‐ and Ctrl‐LFs stained with MitoTracker Green suggest that mitochondrial mass is also increased in IPF‐LFs (Figure 2C). In addition, genes involved in regulating mitochondrial synthesis (PGC‐1α) or components of the mitochondrial electron transport chain (UQCRC2 and NDUFB8) were up‐regulated in IPF‐LFs (Figure 2D), concomitantly with an increase in the activity of mTORC1, a pivotal mediator of mitochondrial homoeostasis (Figure 2E).

### 3.3 Etoposide and rotenone induce Ctrl‐LF senescence

**Figure 3:**
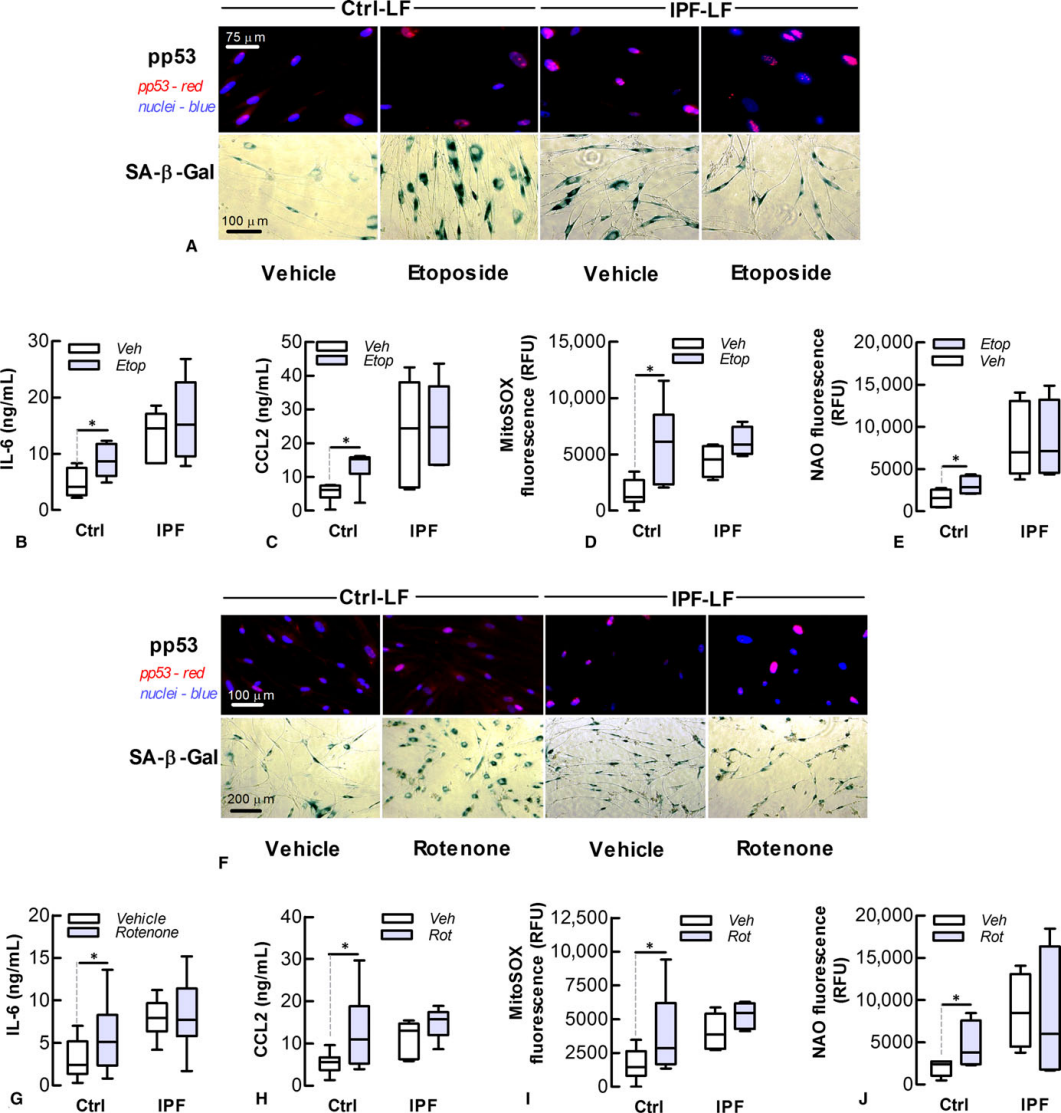
Etoposide and rotenone induce senescence of control lung fibroblasts. IPF‐ and Ctrl‐LFs treated with etoposide (Etop, 10 μmol L^−1^, top panel) or rotenone (Rot, 100 nmol L^−1^, bottom panel) for 72 hour were evaluated for markers of senescence and mitochondrial superoxide production. (A) *Top* Fluorescence analysis of phosphorylated p53 (pp53, red) and nuclei (blue) in etoposide‐treated cells. *Bottom* Cytochemical staining of SA‐β‐Gal (blue). (B‐E) Levels of IL‐6 and CCL2 protein, mitochondrial superoxide and stress of etoposide‐treated cells. *P < 0.05 (n = 4‐7). (F) *Top* Fluorescence images of phosphorylated‐p53 (red) in nuclei (blue) of LFs of rotenone‐treated cells. *Bottom* SA‐β‐Gal cytochemical staining. (G‐J) Levels of IL‐6 and CCL2 protein, mitochondrial superoxide and stress of rotenone‐treated cells. *P < 0.05 (n = 5‐8)

We next investigated the effects of etoposide on the senescent phenotype and mitochondrial function of human LFs. This DNA topoisomerase II inhibitor triggers senescence by causing DNA breaks by interfering with DNA unwinding and subsequent re‐ligation. After a 72‐hour incubation with etoposide (10 μmol L^−1^) in serum‐reduced medium (0.4% v/v FCS), Ctrl‐LFs developed characteristics of senescence including increased formation of p53 nuclear foci, SA‐β‐Gal activity and SASP cytokine production (Figure 3A‐C). Senescence induction was accompanied by increases in mitochondrial superoxide production and stress (Figure 3D‐E). In contrast, etoposide had little effect on the senescent phenotype of IPF‐LFs (see also Figure S2). To investigate whether mitochondrial‐derived ROS contributes to senescence, LF s under similar conditions were also incubated with rotenone (100 nmol L^−1^), which binds the mitochondrial ETC to induce superoxide production. Ctrl‐LFs exhibited a sharp increase in superoxide levels within 60 minute of rotenone treatment, although there was no evidence of an increase in the DDR (i.e, increased nuclear pp53) within the first 6 hour of treatment (Figure S3). However, Ctrl‐LFs showed characteristics of senescence and an increase in the DDR after 72‐hour rotenone incubation (Figure 3F‐H). Concomitantly, rotenone induced long‐term increases in mitochondrial superoxide production and stress (Figure 3I and J). Like etoposide, rotenone had little effect on senescence markers in IPF‐LFs (Figure 3).

### 3.4 NAC suppresses etoposide‐induced senescence

**Figure 4:**
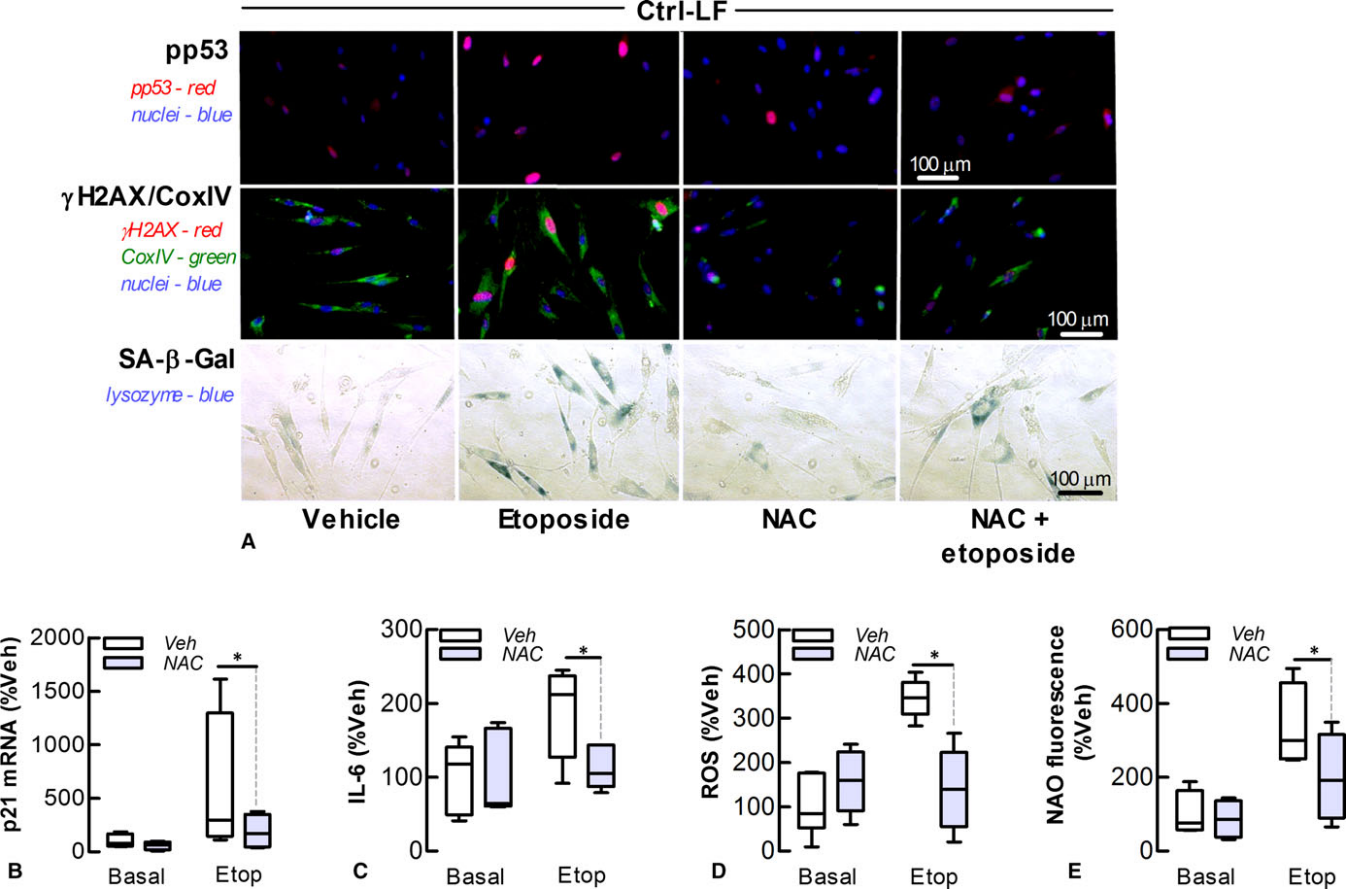
NAC attenuates etoposide‐induced senescence of control lung fibroblasts. The effect of the antioxidant NAC (2 mmol L^−1^) on senescence and mitochondrial homoeostasis in Ctrl‐LFs following incubation with etoposide (Etop, 10 μmol L^−1^) for 72 hour. (A) Fluorescence images *Top* Phosphorylated‐p53 (red) in nuclei (blue) of LFs. *Middle* Phosphorylated‐γH2A.X (red), CoxIV (green) and nuclei (blue). *Bottom* Cytochemical staining of SA‐β‐Gal (blue). (B) Levels of p21 mRNA. (C) Levels of IL‐6 in conditioned media. (D, E) Cytosolic ROS (DCF fluorescence) and mitochondrial stress as measured by fluorometry. *P < 0.05 (n = 4‐6)

The role of ROS in LF senescence was investigated using N‐acetyl cysteine (NAC). This antioxidant replenishes intracellular stores of glutathione, the primary endogenous antioxidant of cells that neutralize excess ROS. NAC (2 mmol L^−1^) attenuated the stimulatory effects of etoposide on senescent markers in Ctrl‐LFs and formation of phosphorylated‐p53 (Figure 4A‐C). Etoposide also increased the number of phosphorylated‐γH2A.X nuclear foci in a NAC‐sensitive manner (Figure 4A). NAC antioxidant activity was shown by its attenuation of etoposide‐induced increases in intracellular ROS production (Figure 4D). NAC also suppressed etoposide‐induced increases in mitochondrial stress and mass as shown by mitochondrial staining with NAO (Figure 4E) and Cox IV antibody (Figure 4A), respectively.

### 3.5 Rapamycin attenuates etoposide‐induced Ctrl‐LF senescence

**Figure 5:**
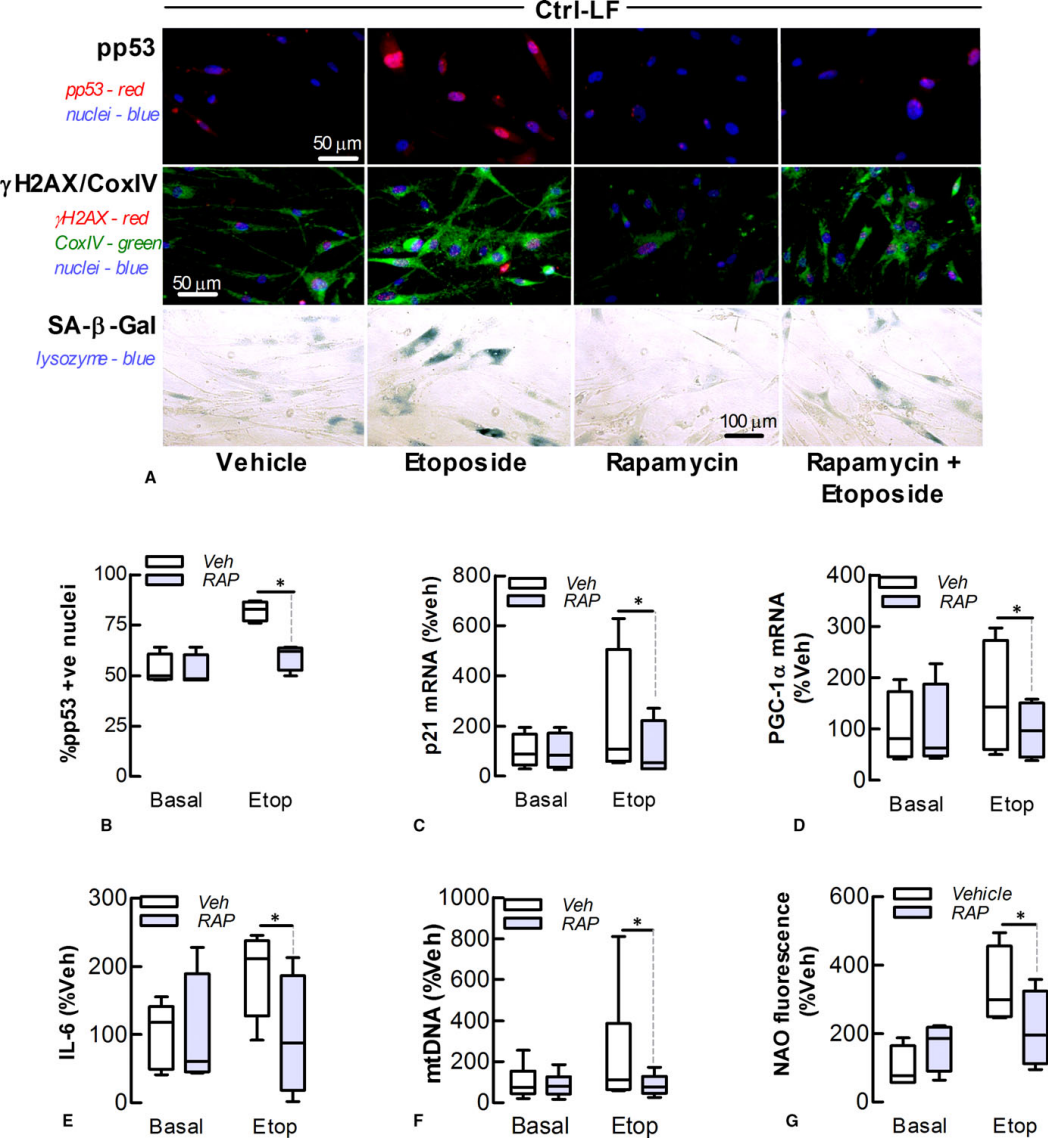
Rapamycin attenuates etoposide‐induced senescence of control lung fibroblasts. The effect of rapamycin (Rap, 0.1 mol L^−1^) on senescence and mitochondrial homoeostasis of Ctrl‐LFs following incubation with etoposide (Etop, 10 μmol L^−1^) for 72 hour. (A) Immunofluorescence. *Top* Phosphorylated‐p53 (red) in nuclei (blue). *Middle* Phosphorylated‐γH2A.X (red), CoxIV (green) and nuclei (blue). *Bottom* Cytochemical staining of SA‐β‐Gal (blue). (B) Quantitation of phosphorylated p53 in nuclei. (C, D) Levels of p21 and PGC‐1α mRNA. (E) Levels of IL‐6. (F, G) Levels of mitochondrial DNA and stress as measured by PCR and fluorometry, respectively. *P < 0.05 (n = 4‐5)

Our data suggest that alterations in mitochondrial homoeostasis involving increased superoxide production have a causative role in LF senescence. To investigate further this possibility, we used rapamycin, the pharmacological inhibitor of mTORC1, which has a pivotal role in regulating mitochondrial biogenesis and activity, including PGC‐1α expression. Pre‐treatment of Ctrl‐LFs with rapamycin (100 nM) attenuated the effects of etoposide on senescent markers, PGC‐1α gene expression and mitochondrial stress, mass and DNA levels (Figure 5).

### 3.6 Rapamycin and mitoTEMPO decelerate IPF‐LF senescence

**Figure 6:**
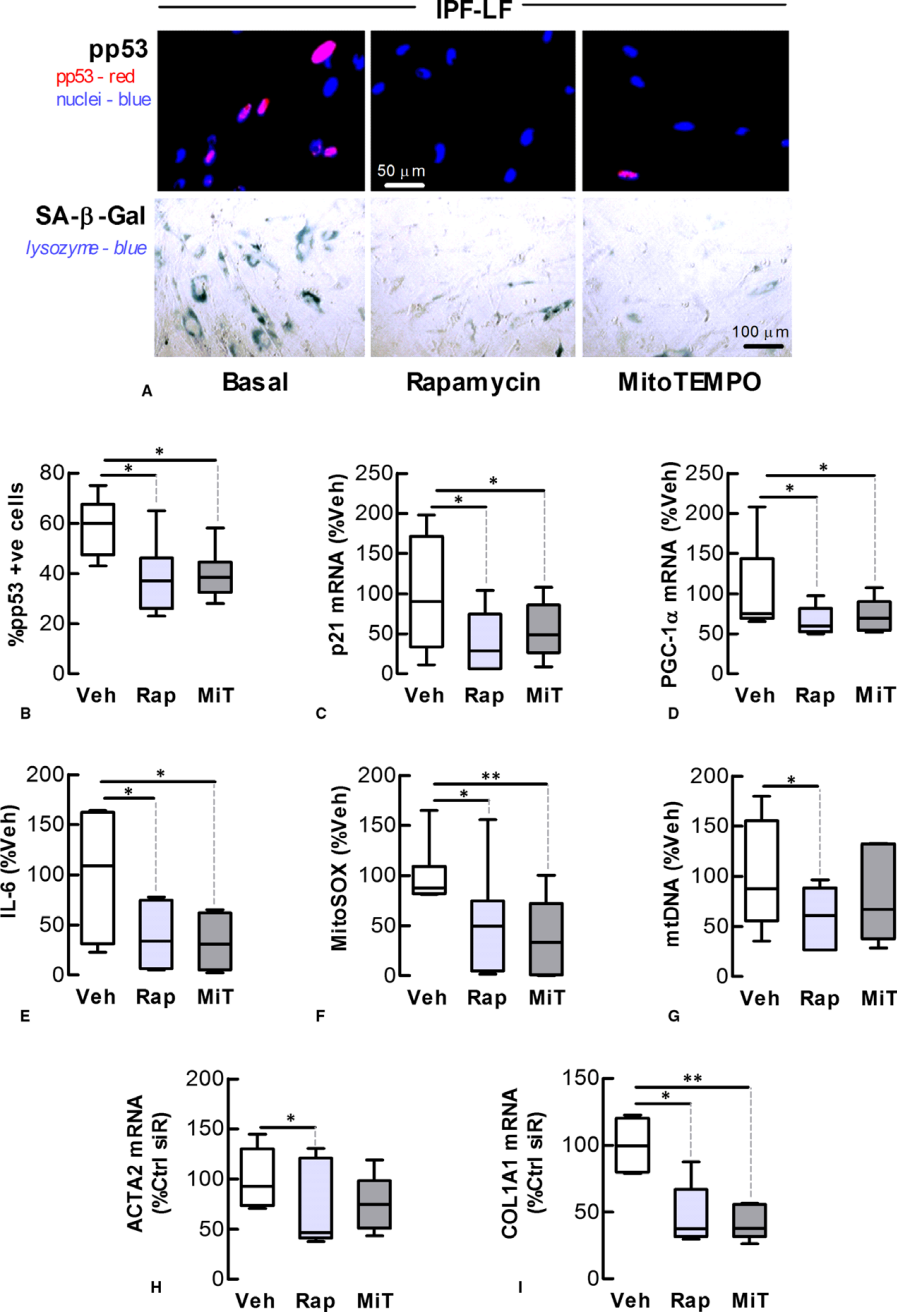
Rapamycin and mitoTEMPO decelerate IPF lung fibroblast senescence. The effect of rapamycin (Rap, 0.1 mol L^−1^) and mitoTEMPO (MiT, 1 mol L^−1^) on senescence and mitochondrial homoeostasis of IPF‐LFs for 8 days. (A) Immunofluorescence. *Top* Phosphorylated‐p53 (red) in nuclei (blue). *Bottom* Cytochemical staining of SA‐β‐Gal (blue). (B) Quantitation of phosphorylated p53 in nuclei. (C, D) Levels of p21 and PGC‐1α mRNA. (E) Levels of IL‐6. (F, G) Levels of mitochondrial superoxide and DNA, as measured by fluorometry and PCR, respectively. (H, I) Levels of α‐SMA (ACTA2) and collagen type I α1 (COL1A1) mRNA. *P < 0.05, **P < 0.01 (n = 5‐7)

We next investigated the effects of long‐term treatment with rapamycin and mitoTEMPO, a mitochondrial‐selective antioxidant, on IPF‐LF senescence. IPF‐LFs maintained in 10% serum containing medium, initially at lower densities to allow for proliferation over an extended period, were incubated with rapamycin (100 nmol L^−1^) or mitoTEMPO (MiT, 1 μmol L^−1^). The media and pharmacological agents were replenished every 2‐3 days for 8 days. Rapamycin or mitoTEMPO treatment reduced markers of senescence, the DDR and SASP cytokines (Figure 6A‐D), accompanied by reductions in mitochondrial superoxide production and DNA content (Figure 6E‐G). MitoTEMPO also attenuated etoposide‐induced increases in senescence marker expression and levels in cultures of Ctrl‐LFs (Figure S4).

### 3.7 Rapamycin and mitoTEMPO down‐regulate myofibroblast marker expression

Senescent LFs exhibit myofibroblast‐like characteristics, including increased levels of α‐smooth muscle actin (α‐SMA).^12^, ^24^ In this study, etoposide stimulated increases in the levels of mRNA encoding the myofibroblast markers, α‐SMA and collagen type I α1 in Ctrl‐LFs (Figure S5). Furthermore, rapamycin and mitoTEMPO attenuated the effects of etoposide (Figure S5), and reduced the expression of myofibroblast markers in IPF‐LFs (Figure 6H‐I).

### 3.8 PGC‐1α knockdown decelerates IPF‐LF senescence

**Figure 7:**
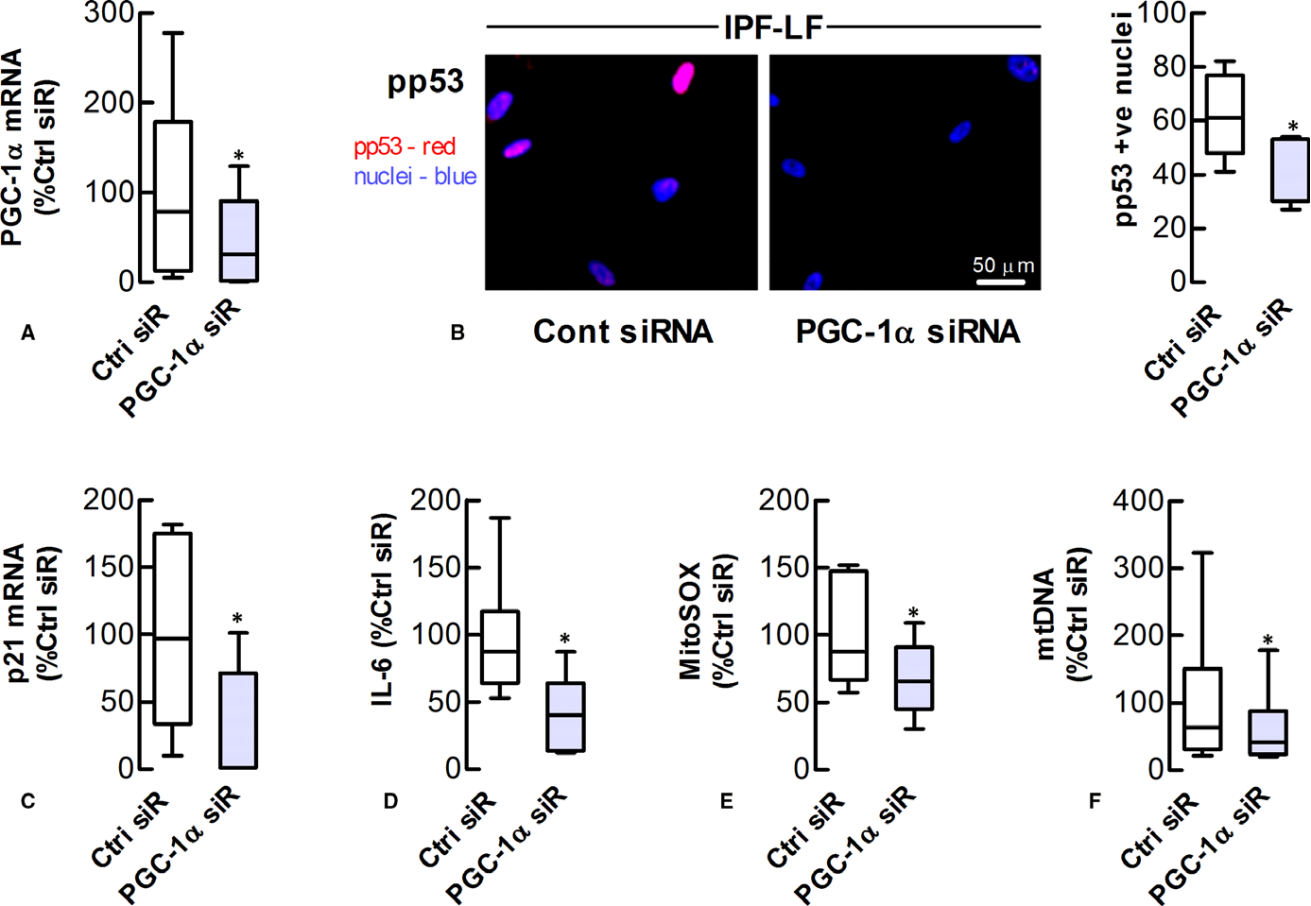
PGC‐1α knockdown decelerates IPF lung fibroblast senescence. The effect of PGC‐1α siRNA (siR) transfection on senescence and mitochondrial homoeostasis of IPF‐LFs for 8 days. (A) Levels of PGC‐1α mRNA in transfected cells (B) *Left* Immunofluorescence images showing phosphorylated‐p53 (red) in nuclei (blue). *Right* Quantitation of phosphorylated‐p53 in nuclei. (C) Levels of p21 mRNA. (D) Levels of IL‐6. (E, F) Levels of mitochondrial superoxide and DNA, as measured by fluorometry and PCR, respectively. *P < 0.05 (n = 6)

As PGC‐1α is a primary regulator of mitochondrial biogenesis and activity downstream of mTORC1, we also explored the impact of PGC‐1α knockdown on IPF‐LF senescence. Transfection of IPF‐LFs maintained in 10% serum containing medium with PGC‐1α‐targeting siRNA 3 times over an 8‐d period reduced markers of senescence, accompanied by decreases in the levels of mitochondrial superoxide and DNA (Figure 7). Immunoblotting showed PGC‐1α siRNA transfection reduced the levels of a low molecular weight (MW) form of PGC‐1α (~50 kDa), which was the most abundant of the PGC‐1α subtypes detected in LFs (Figure S6). In the absence of transfection, baseline levels of this PGC‐1α variant were higher in cultures of IPF‐LFs than Ctrl‐LFs (Figure S7).

## 4 DISCUSSION

The mechanisms that underlay senescence in lung fibroblasts, including the specific contribution of mitochondria, remain unclear. In this study, IPF‐LFs exhibited an array of senescence‐like characteristics in vitro, including an increased DDR and SASP. These were accompanied by alterations in mitochondrial homoeostasis, as evidenced by enhanced superoxide production, increased levels of mtDNA and activation of mTORC1. The DNA damaging agent, etoposide induced senescence in Ctrl‐LFs that was associated with mitochondrial perturbation. However, etoposide had insignificant effect on IPF‐LF phenotype, possibly because a high proportion of these cells were already senescent. Pharmacological‐induced senescence was sensitive to antioxidant treatment, suggesting a role of ROS in the development of LF senescence. Supporting this finding, mitochondrial perturbation and increased superoxide production induced by rotenone mimicked the senescence‐inducing effects of etoposide in Ctrl‐LFs. Furthermore, pharmacological inhibition of the mitochondrial regulator, mTORC1 attenuated chemical‐induced senescence in Ctrl‐LFs, and decelerated IPF‐LF senescence in long‐term culture. Promisingly, the mitochondrial‐selective antioxidant, mitoTEMPO, had a comparable effect on IPF‐LF senescence. Overall, this study provides substantial evidence that senescence and mitochondrial dysfunction are highly interrelated processes in IPF‐LFs and that targeting either mitochondrial‐derived superoxide or the DDR‐driven processes that contribute to its increased production may limit the damaging impact of senescence in IPF.

The IPF‐LFs in this study displayed many of the senescence characteristics reported previously, including increased p16 and p21 expression and SA‐β‐Gal activity.^12^, ^24^ In addition, for the first time to our knowledge, IPF‐LFs were associated with increased nuclear activation of p53. As part of the DDR, ataxia‐telangiectasia mutated kinase (ATM) phosphorylates p53 at sites of DNA double‐stranded breaks (DSBs).^5^ Nuclear activation of p53 correlates with DSB formation and the subsequent DDR. In this study, IPF‐LFs exhibited a higher proportion of nuclei containing foci of phosphorylated p53. In DDR signalling, activated p53 up‐regulates p21 gene expression and levels, which in turn mediates cell‐cycle arrest through cyclin‐dependent kinase (CDK) inhibition. While speculative and difficult to show, increased numbers of senescent LFs may exist in IPF patients before the onset of disease, with a primary role in IPF pathogenesis. These senescent LFs would amplify aberrant wound repair processes that follow epithelial injury, exacerbating the fibrotic response. Factors contributing to the heightened LF senescence that precedes IPF development include age, genetics, oxidative stress (involving mitochondrial dysfunction) and lung injuries. Higher levels of LF senescence may also be a consequence of secondary events in IPF pathology, such as the replicative senescence that occurs during the fibroproliferative phase of the disease. Senescence can also spread from senescent to naïve fibroblasts in the ageing fibrotic lung as a consequence of the “bystander effect”.^28^ Regardless of the reason(s) for increased IPF‐LF senescence, these cells are likely to have important roles in IPF because of their resistance to apoptosis, myofibroblast‐like features and development of a SASP.

The SASP is an important pathological feature of senescence.^6^, ^29^ In this study, IPF‐LFs exhibited increased production of cytokines typically associated with the SASP, as well as a markedly pro‐inflammatory gene expression profile. Profiling revealed the expression of several transcription factors was higher in IPF, including NF‐κB and C/EBPβ, important mediators of the secretory component of senescent cells.^30^ In senescence, NF‐κB signalling follows the DDR via activation of p38 MAPK and its downstream mediator, MAPKAPK2, both of which were up‐regulated in IPF‐LFs.^30^ Furthermore, the expression of two key modulators of the innate immune system that regulate NF‐κB activation, MyD88 and toll‐interacting protein (Tollip), was also higher in IPF‐LFs. Evidence is accumulating that chronic low‐level activation of the innate immune system has an important role in IPF, with several Tollip gene polymorphisms linked with the disease.^31^ A negative regulator of acute inflammation, Tollip also augments chronic low‐grade inflammation in a process that involves its translocation to mitochondria.^32^ Many of the inflammatory gene changes reported in this study align with our current understanding of IPF pathology and/or processes of cellular senescence.

Mitochondrial dysfunction is a feature of both senescence and ageing.^19^ Mitochondrial DNA, membranous lipids and proteins are highly susceptible to oxidative damage, causing respiratory uncoupling and increased formation of superoxide.^33^ The latter in turn contributes to further mitochondrial damage, as part of a cycle that ultimately leads to dysmorphic and impaired mitochondria. In this study, IPF‐LF mitochondrial dysfunction was shown by increases in MitoSOX and NAO fluorescence, which were not evident in Ctrl‐LFs. The MitoSOX probe accumulates in mitochondria where it selectively binds superoxide, whereas NAO binds to the depolarized mitochondrial inner membrane, serving as an indicator of mitochondrial stress. Levels of the Nox4 enzyme, which also forms superoxide, are increased in IPF‐LFs, and Nox4 is implicated in fibroblast senescence in pulmonary fibrosis.^11^ Dysregulated Nox4 may contribute to IPF‐LF mitochondrial dysfunction, particularly as Nox4 can localize in mitochondria, and its expression is stimulated by mitochondrial‐derived ROS.^31^ Another important pathological feature of mitochondrial dysfunction is the cellular release of fragmented, oxidised mtDNA, which acts as danger associated molecular pattern (DAMP).^33^ IPF‐LFs in culture, when compared to Ctrl‐LF, release increased amounts of mtDNA.^34^ Furthermore, BALF and serum levels of mtDNA are higher in patients with IPF, as compared to control donors, with serum levels being predictive of all‐cause mortality.^34^

In addition to respiratory decoupling, elevated levels of mitochondrial superoxide in IPF‐LFs could also be a consequence of increases in mitochondrial mass. The dysregulation of mitochondrial biogenesis, fusion, fission and mitophagy all contribute to changes in the mitochondrial pool in ageing and disease.^19^, ^23^, ^34^, ^35^ Increased mitochondrial mass is reported in AECII and lung fibroblasts in IPF and airway smooth muscle (ASM) cells in severe asthma.^23^, ^36^, ^37^ In this study, increased levels of mtDNA and MitoTracker Green staining suggest IPF‐LFs also exhibit a net increase in mitochondrial mass as compared to Ctrl‐LFs, possibly a consequence of increased biogenesis. In support of this hypothesis, the IPF‐LFs showed an increase in mTORC1 activity and PGC‐1α expression. PGC‐1α is a key regulator of mitochondrial biogenesis and is directly up‐regulated by mTORC1 at a transcriptional level. Impaired recycling of mitochondria (mitophagy) may also explain our findings of increased mitochondrial mass in IPF‐LF cultures. While not investigated in this study, mitophagy has been reported to be suppressed in IPF‐LFs.^38^ Contrary to our observations, Rojas and colleagues reported a decrease in the mitochondrial mass of fibroblasts isolated from IPF lung.^24^ Variations in fibroblasts isolation and culture conditions between these two studies may contribute to these divergent findings. In particular, our fibroblast culture protocol used a high glucose containing medium favouring increased mitochondrial biogenesis. 5′ AMP‐activated protein kinase (AMPK) is activated when energy stores are low to regulate mitochondrial biogenesis by inhibiting mTORC1,^39^ which may have been relevant in the Rojas study.

In this study, we provide evidence of a causal link between LF senescence and mitochondrial dysfunction. mTORC1 is a key mediator of cellular senescence, which links the DDR with alterations in mitochondrial homoeostasis in an auto‐amplifying loop that perpetuates senescence.^40^ As part of this loop, ATM via AKT activates mTORC1, which regulates mitochondrial biogenesis through PGC‐1α/β expression and by inhibiting eukaryotic translation initiation factor 4E (eIF4E)‐binding proteins (4E‐BPs).^41^ Furthermore, mTORC1 suppresses mitophagy and the expression of the antioxidant enzyme, mitochondrial superoxide dismutase (MnSOD).^38^ Inhibition of the mTORC1 pathway is reported to attenuate γ‐irradiation‐induced senescence in fibroblasts by suppressing mitochondrial biogenesis and subsequent superoxide production.^23^ In this study, rapamycin attenuated etoposide‐induced Ctrl‐LF senescence and decelerated IPF‐LF senescence in long‐term culture. Furthermore, the knockdown of PGC‐1α in IPF‐LFs resulted in a marked reduction in expression of senescence markers. Overall, these observations provide additional evidence of the importance of mitochondria and mTORC1 signalling in the maintenance of LF senescence. Increased mTORC1 activation within the fibrotic foci of IPF patients suggests this pathway has a role in pulmonary fibrosis.^42^ Recently the anti‐fibrotic potential of the pan‐PI3 kinase/mTOR inhibitor, GSK2126458 was evaluated in ex vivo models of IPF with promising results.^43^ However, rapamycin has also been associated with a more rapid disease progression in a cohort of IPF patients.^44^ As mTORC1 regulates a range of processes outside of mitochondrial homoeostasis, with many potential biological effects, alternative targets may be required to limit the deleterious effects of dysfunctional mitochondria on senescence in the treatment for IPF. In this regard, mitoTEMPO and other mitochondrial‐selective antioxidants such as MitoQ show potential as therapies for lung fibrosis. Here we observed that mitoTEMPO decelerates IPF‐LF senescence in vitro, and in pre‐clinical studies for asthma and kidney disease, mitoTEMPO exhibits anti‐fibrotic activity.^1^, ^45^, ^46^

In this study, the expression of PGC‐1α was increased in IPF‐LFs and senescence‐induced Ctrl‐LFs. However, Yu et al^47^ recently reported that levels of PGC‐1α protein were decreased in lung homogenates of IPF patients (even though levels of its mRNA were increased). The PGC‐1α detected in lung lysates by immunoblotting was comparable in size to the PGC‐1α1 isoform, which has a predicted MW of 92 kDa, with lower MW forms not described or shown. Here, the predominant form of PGC‐1α detected in LFs had a lower apparent MW, between 50 and 75 kDa. While PGC‐1α1 is the most widely characterised PGC‐1α isoform, there are numerous lower MW versions, including PGC‐1α2 and PGC‐1α3, both of which are closer to 50 kDa in size.^48^ These splice variants are regulated differently to PGC‐1α1 and evoke distinct biological programs.^49^ An important function ascribed to PGC‐1α, asides from its role in mitochondrial biogenesis, is the induction of antioxidant expression.^50^ The antioxidant role of PGC‐1α is dependent on the energy status of the cell and involves activation of its upstream regulator AMPK under conditions of energetic stress.^50^ However, the knockdown of PGC‐1α in IPF‐LFs under the conditions used in this study was not only associated with reduced levels of mitochondrial DNA, but also superoxide generation. These data suggest that PGC‐1α‐dependent antioxidant responses in IPF‐LFs are not sufficient to overcome increased ROS production as a consequence of PGC‐1α‐dependent mitochondrial biogenesis. These observations may in part be a result of alternative splicing patterns and/or post‐translational modifications of PGC‐1α in IPF‐LFs. While the PGC‐1α knockdown data of this study supports a role of mitochondrial biogenesis in the reinforcement of LF senescence, further investigation is required to ascertain the specific contribution of PGC‐1α and its various isoforms in lung fibrosis.

In this study, we provide evidence that an auto‐feedback loop exists between dysfunctional mitochondrial and senescence in LFs, involving increases in mitochondrial superoxide and mTORC1 activation. However, there are inherent difficulties in confirming the existence of such a loop, particularly as senescence and mitochondrial dysfunction have a range of overlapping pathological features and effects. Another contributing factor is the non‐selectivity of the tools that can be utilized with primary cultures of LFs to delineate the role of mitochondria in the acquisition and reinforcement of senescence. We have made mention of the limitations of the approaches employed to target mitochondrial function and biogenesis, including the blunt effects of rapamycin and PGC‐1α knockdown. To date, one of the more convincing studies to show the important contribution of mitochondria to senescence was by Passos and colleagues, who used recombinant genetics to remove mitochondria from embryonic fibroblasts (ie, MRC‐5s).^23^ However, to conduct such mechanistic studies is less feasible with primary cultures of LFs from aged donors/patients. What our studies do confirm is that mitochondrial dysfunction involving increased superoxide occurs in parallel with senescence in IPF‐LFs. These processes are highly intertwined and that a range of mitochondrial targeting approaches attenuates/decelerates senescence.

In summary, LFs isolated from IPF patients exhibit senescence‐like characteristics in association with an increased DDR, SASP and changes in mitochondrial homoeostasis. Our data also support roles of increased mitochondrial stress and superoxide production in the induction and maintenance of the senescent phenotype in LFs. The involvement of dysfunctional mitochondria in LF senescence may provide alternate targeting opportunities in the treatment of IPF.

## AUTHOR CONTRIBUTION

DAK, MS and DVP involved in concept and design; MS, JR, DVP, DWW, KECB, ATR, CMH, NK, JJ and GW performed acquisition, analysis and interpretation of data; and DAK, MS, CG, JKB, CMP and SEM performed drafting the manuscript for intellectual content.

## CONFLICTS OF INTEREST

The authors confirm that there are no conflicts of interest.
