# Supplementary material for: Mitochondrial dysfunction contributes to the senescent phenotype of IPF lung fibroblasts
Source: J Cell Mol Med. 2018 Sep 26;22(12):5847–61. doi: 10.1111/jcmm.13855 (PMC6237609; doi:10.1111/jcmm.13855)
Supplement: Supplementary file 1 [file JCMM-22-5847-s001.docx]

**Mitochondrial dysfunction contributes to the senescent phenotype of IPF lung fibroblasts**

Michael Schuliga, Dmitri V Pechkovsky, Jane Read, David W Waters, Kaj EC Blokland, Andrew T Reid, Cory M Hogaboam, Nasreen Khalil, Janette K Burgess, Cecilia M Prêle, Steven E Mutsaers, Jade Jaffar, Glen Westall, Christopher Grainge and Darryl A Knight

***Online supplement***

**Tables**

| **No.** | **Sex** | **Age** | **Smoking**  **history** | **FEV1 %pred** | **FVC**  **%pred** | **TLCO %pred** |
| --- | --- | --- | --- | --- | --- | --- |
| 1 | M | 62 | Never | 45 | 37 | n/a |
| 2 | M | 61 | Ex (8y) | 49 | 41 | 50 |
| 3 | M | 65 | Ex (13y) | 48 | 43 | n/a |
| 4 | M | 69 | Ex (24y) | 86 | 86 | 17 |
| 5 | M | 63 | Ex (14y) | n/a | n/a | 78 |
| 6 | F | 56 | Never | 53 | 47 | 16 |
| 7 | M | 57 | Ex (5y) | 50 | 42 | 13 |
| 8 | F | 59 | Ex (2y) | 68 | 58 | n/a |
| 9 | M | 61 | Ex (18y) | 64 | 56 | n/a |
| 10 | M | 43 | Ex (2y) | 39 | 33 | 40 |
| 11 | M | 67 | Never | 43 | 38 | 43 |
| 12 | F | 71 | Never | 41 | 39 | 60 |
| 13 | F | 66 | Never | 67 | 60 | 67 |
| 14 | M | 73 | Ex (47y) | n/a | n/a | n/a |
| 15 | n/a | n/a | n/a | n/a | n/a | n/a |

**Table S1.** **Characteristics of IPF patients**. Characteristics include gender, age, smoking history (years since stopped), forced expiratory volume in 1 second (FEV_1_) %predicted, forced vital capacity (FVC) % predicted and transfer factor of the lung for carbon monoxide (TLCO). n/a (not assessed or available).

| **Figure** | **Ctrl-LFs** | | | **IPF-LFs** | |
| --- | --- | --- | --- | --- | --- |
| 1a | | N=5 (2), 57 y | N=6 (0), 63.4 y | |  |
| 1b | | N=7 (1), 53.7 y | N=7 (0), 58.4 y | |  |
| 1c | | N=8 (1), 59.4 y | N=10 (0), 61.9 y | |  |
| 1d | | N=5 (2), 66.7 y | N=6 (0), 61.0 y | |  |
| 1e (Ccl2) | | N=7 (3), 56.8 y | N=9 (1), 65.5 y | |  |
| 1e (Ccl5) | | N=6 (2), 57.0 y | N=8 (1), 64.4 y | |  |
| 1e (IGFBP-5) | | N=6 (2), 57.0 y | N=8 (1), 64.4 y | |  |
| 1e (IL-6) | | N=7 (2), 59.2 y | N=8 (1), 64.4 y | |  |
| 1e (IL-8) | | N=6 (2), 57.0 y | N=8 (1), 64.4 y | |  |
| 1f | | N=6 (2), 59.0 y | N=5 (0), 63.4 y | |  |
| 2a (Superoxide) | | N=7 (2), 62.8 y | N=6 (1), 63.4 y | |  |
| 2a (ROS) | | N=5 (2), 65.3 y | N=4 (0), 63.5 y | |  |
| 2a (Stress) | | N=5 (2), 61.0 y | N=5 (0), 62.2 y | |  |
| 2a (mtDNA) | | N=5 (0), 55.8 y | N=7 (0), 57.7 y | |  |
| 2d (PGC-1α) | | N=6 (2), 57.0 y | N=7 (0), 57.7 y | |  |
| 2d (PGC-1β) | | N=4 (2), 56.5 y | N=4 (0), 65.3 y | |  |
| 2d (NDUFB8) | | N=5 (2), 69.3 y | N=5 (0), 64.6 y | |  |
| 2d (UQCRC2) | | N=5 (2), 69.3 y | N=5 (0), 64.6 y | |  |
| 2e | | N=7 (2), 59.2 y | N=6 (0), 63.7 y | |  |
| 3b | | N=5 (1), 67 y | N=5 (0), 66.8 y | |  |
| 3c | | N=6 (1), 67.8 y | N=5 (0), 66.8 y | |  |
| 3d | | N=7 (2), 64.8 y | N=4 (0), 61.2 y | |  |
| 3e | | N=5 (2), 61.0 y | N=5 (0), 63.2 y | |  |
| 3g | | N=8 (1), 56.0 y | N=6 (0), 63.5 y | |  |
| 3h | | N=7 (2), 62.4 y | N=5 (0), 63.4 y | |  |
| 3i | | N=8 (3), 60.0 y | N=5 (0), 63.0 y | |  |
| 3j | | N=5 (2), 61.0 y | N=5 (0), 63.2 y | |  |

**Table S2. Age matching of patients and donors for IPF-LF versus Ctrl-LF experiments.** Average ages of patients and donors (those known) for the Ctrl- and IPF-LFs respectively, of the listed experiments. The number in brackets is the number of patients or donors for which age was not available.

| **Gene** | **Forward primer** | | **Reverse primer** | |  |
| --- | --- | --- | --- | --- | --- |
| 18S ribosomal RNA  (18S rRNA) | | ATCGGGGATTGCAATTATTC | | CTCACTAAACCATCCAATCG | |
| ACTA2  (α-smooth muscle actin) | | AGATCAAGATCATTGCCCC | | TTCATCGTATTCCTGTTTGC | |
| β2-microglobulin | | TGCTGTCTCCATGTTTGA TGTATCT | | TCTCTGCTCCCCACCTCTAAGT | |
| COL1A1  (Collagen type I α1) | | TCATCTCCATTCTTTCCAGG | | GCTATGATGAGAAATCAACCG | |
| CDKN1A (p21) | | CAGCATGACAGATTTCTACC | | CAGGGTATGTACATGAGGAG | |
| CDKN2A (p16) | | AGCATGGAGCCTTCG | | ATCATGACCTGGATCGG | |
| GAPDH | | CTT TTG CGT CGC CAG | | TTGATGGCAACAATATCCAC | |
| NDUFB8 | | CTTGGCATGTCATGTGTATG | | TAAGGATACTGCTTTGGTCC | |
| PPARGC1A (PGC-1α) | | GCAGACCTAGATTCAAACTC | | CATCCCTCTGTCATCCTC | |
| PPARGC1B (PGC-1β) | | ACATTCAAAATCTCTCCAGC | | CTCTCCTATTTCTTGTCAGC | |
| tRNA-Leu (UUR) | | CACCCAAGAACAGGGTTTGT | | TGGCCATGGGTATGTTGT TA | |
| UQCRC2 | | GTGAGTCATCCTGTTCTA AAG | | CATTCTGTTCTCGGATTTCAC | |

**Table S3. PCR primers**

**Figures**

**Figure S1** *Lung fibroblasts of IPF patients exhibit a SASP.* **(a)** Levels of six differentially regulated genes of the nCounter human inflammatory gene panel. **(b)** *Top* Immunoblot detection of NF-κB p65/RelA in cell lysates obtained from LFs of separate IPF and Ctrl donors. *Bottom* Fluorescent detection of total protein in the gel before immunoblotting to verify protein loading.

**Figure S2.** *Etoposide induces increased detection of phosphorylated-γH2A.X nuclei foci in control lung fibroblasts.* IPF- and Ctrl-LFs treated with etoposide (Etop, 10 μM, top panel) for 72 h. Representative immunofluorescence images of phosphorylated γH2A.X (red) and nuclei (blue) in IPF- and Ctrl-LFs.

**Figure S3.** *Rotenone induces rapid increases in mitochondrial superoxide production by Ctrl-LFs.* LFs treated with rotenone (0.1 μM) were evaluated for mitochondrial superoxide production and markers of senescence. **(a, b)** MitoSOX fluorescence (red) in Ctrl-LFs treated with rotenone for 0.5 to 4 h. **(c)** Fluorescence analysis of phosphorylated p53 (red) in nuclei (blue) of Ctrl-LFs treated with rotenone or etoposide (10 μM) for 3 to 6 h. *P<0.05 (n=5).

**Figure S4.** *MitoTEMPO attenuates etoposide-induced senescence of control lung fibroblasts.* The effect of the mitoTEMPO (MiT, 1 μM) on senescence and mitochondrial homeostasis in Ctrl-LFs following incubation with etoposide (Etop, 10 μM) for 72 h. **(a)** Fluorescence images *Top* Phosphorylated-γH2A.X (red) and nuclei (blue). *Bottom* Cytochemical staining of SA-β-Gal (blue). (**b, c**) Levels of p21 and PGC-1α mRNA.

**Figure S5.** *Rapamycin and mitoTEMPO attenuate etoposide-induced in increases in a-SMA and collagen type Ia1 gene expression in control lung fibroblasts.* The effect of rapamycin (Rap, 0.1 μM) **(a-b)** and mitoTEMPO (MiT, 1uM) **(c-d)** on Ctrl-LF gene expression following incubation with etoposide (Etop, 10 μM) for 72 h. **(a, c)** Levels of ACTA2 (α-SMA) and **(b, d)** COL1A1 (collagen type Iα1) mRNA.

**Figure S6** *PGC-1α siRNA transfection decreases the levels of a low molecular weight form of PGC-1α.* **(a, b)** *Top* Immunoblot detection of PGC-1α in cell lysates of IPF-LFs 48 h after control (ctrl) and PGC-1α siRNA transfection. The left (a) and right (b) blots were probed using anti-PGC-1 α(/β) IgG from Cell Signaling Technology (2178S) and Abcam (ab72230) respectively. *Bottom* Immunoblot detection of β-actin (Abcam, ab8227) to verify protein loading. All antibodies were used at 1:1000 according to the protocol described in the main manuscript.

**Figure S7** *Lung fibroblasts of IPF patients exhibit higher levels of PGC-1a.* *Top* Immunoblot detection of PGC-1α in cell lysates obtained from LFs of separate IPF and Ctrl donors. The blot was probed using anti-PGC-1α IgG from Cell Signaling Technology (2178S) at 1:2000. *Bottom* Fluorescent detection of total protein in gel before immunoblotting to verify protein loading.

*Figure S1*





*Figure S2*





*Figure S3*


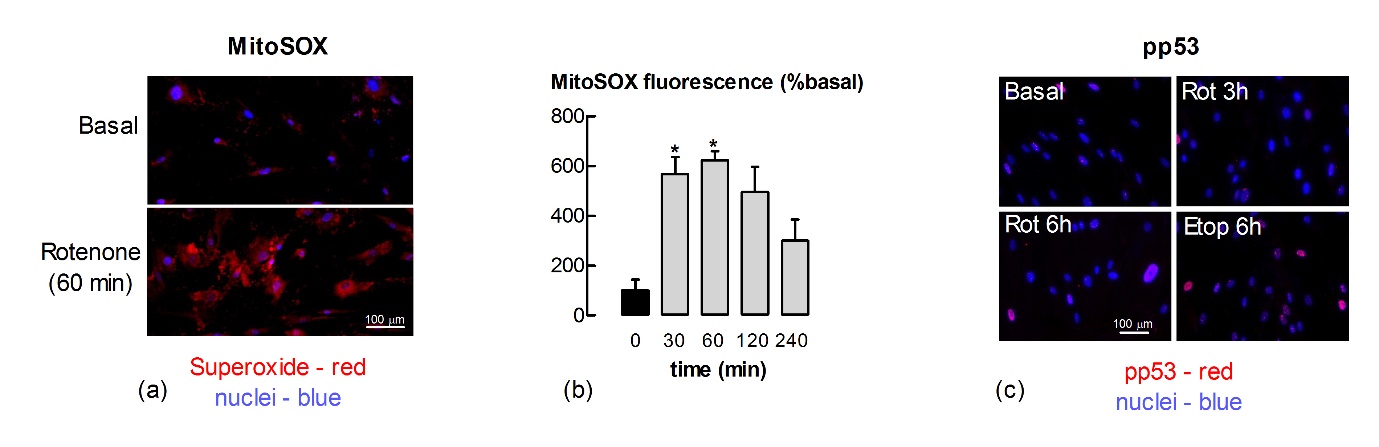


*Figure S4*

*

*

*Figure S5*





*Figure S6*

*

*

*Figure S7*

*

*
